# A Traffic Light System for Detecting Spinal Deformities in Children with Cerebral Palsy

**DOI:** 10.3390/children12101315

**Published:** 2025-09-30

**Authors:** Bettina Westhoff, Björn Vehse, Kell Behrens, Melanie Horter, Reza Pasha, Richard Placzek, Urs von Deimling, Tamara Seidl, Daniel Herz

**Affiliations:** 1 University Hospital, Medical Faculty, Department of Orthopedics and Traumatology, 40225 Düsseldorf, Germany; westhoff@med.uni-duesseldorf.de; 2 DRK Pediatric Hospital, Department of Neuroorthopedics, 57072 Siegen, Germany; 3 Independent Researcher, 53474 Bad Neuenahr-Ahrweiler, Germany; 4 Social Pediatric Center, 48653 Coesfeld, Germany; 5 Helios Klinikum, Department of Orthopedics and Traumatology, 47805 Krefeld, Germany; 6 University Hospital, Medical Faculty, Department of Orthopedics and Traumatology, 53127 Bonn, Germany; 7 Independent Researcher, 53359 Rheinbach, Germany; 8 Klinikum Herford, Department of Orthopedics and Traumatology, 32049 Herford, Germany; 9 Marienstift, Department of Pediatric Orthopedic Surgery, 99310 Arnstadt, Germany

**Keywords:** cerebral palsy, scoliosis, spinal deformity, prevention

## Abstract

**Highlights:**

**What are the main findings?**

- The TLS-Spine is an easy-to-use tool for pediatricians and physiotherapists in daily clinical routine for getting an impression of the condition of the spine in CP children.
- It helps clinicians to perform a clinical examination, to understand the findings and to decide on further management of the patients.

**What is the implication of the main finding?**

- The TLS-Spine may increase the awareness for spinal deformities in CP children and enables early referral to orthopedic specialists for early detection and treatment.

**Abstract:**

**Background/Objectives**: Currently, clinicians and physiotherapists pay only limited attention to spinal deformities in children with cerebral palsy (CP). To enhance awareness, a tool based on a traffic light system (TLS-Spine) was developed for use by pediatricians and physiotherapists caring for children with CP. The objective of the study was to evaluate the applicability of this assessment tool in routine clinical practice. **Methods**: A review group consisting of 48 pediatricians and physiotherapists was recruited. Each participant was asked to apply the TLS-Spine to a minimum of 10 CP patients and to complete a questionnaire concerning its value and applicability in daily practice. Responses were rated on a scale from 1 (complete agreement) to 6 (complete disagreement). **Results**: The TLS-Spine was correctly applied in 96.3% cases. The questionnaires of 48 reviewers based on 537 completed survey sheets were analyzed. Overall, reviewers reported no difficulties with the introduction and use of the TLS-Spine in daily clinical routine practice (median/range: 1/1–4). The tool was considered straightforward to use (1/1–3), not time consuming (1/1–4), helpful in performing the clinical examination (2/1–6), understanding the findings (2/1–6) and deciding on further management of the patients (2/1–5). Physicians rated the TLS-Spine significantly higher than physiotherapists in four of nine dimensions. **Conclusions**: The TLS-Spine is a practical and user-friendly assessment tool. It may increase the awareness for spinal deformities and support early referral to orthopedic specialists for early detection and treatment.

## 1. Introduction

**Figure 1:**
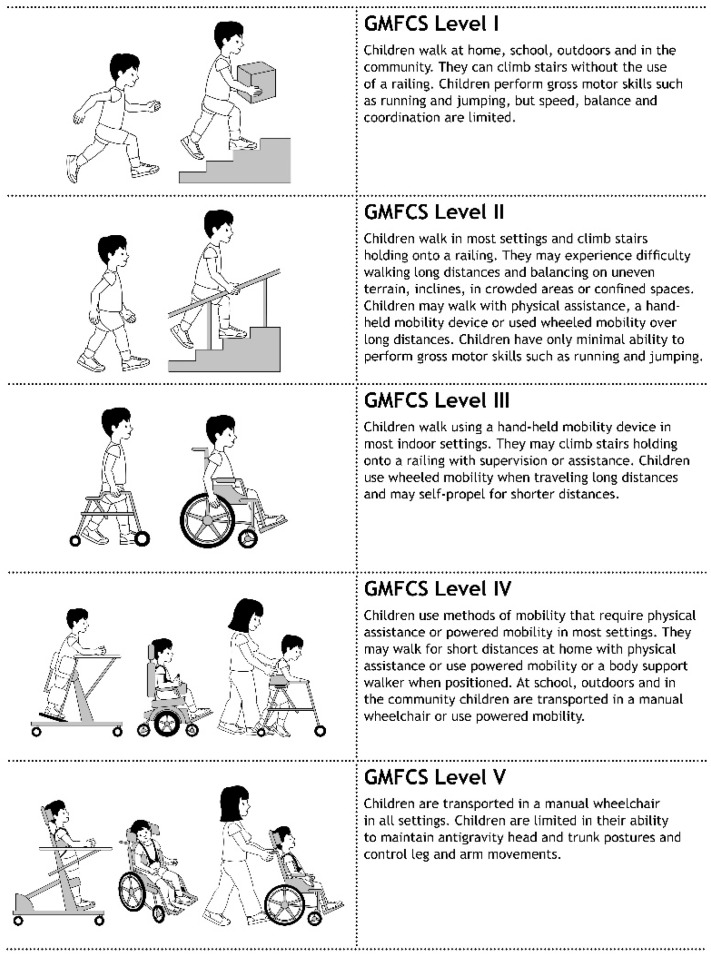
The Gross Motor Function Classification System GMFCS between 6th and 12th birthday by Palisano et al. [2] (Illustrations with kindly permission of Prof. Kerr Graham, Melbourne, Australia).

Cerebral palsy (CP) is a static neurologic condition resulting from brain injury that occurred prior to complete development of the brain during the prenatal, perinatal or postnatal period. It is the most common childhood physical disability and affects 2–3 children per 1000 live births [1]. Apart from other conditions, CP is characterized by impairment of the motor system, including delay in motor development, abnormal muscle tone, muscular weakness and loss of selective motor control, resulting in balance problems, progressive muscle contractures and gait disturbances. Depending on the functional impairments, patients are classified according to the Gross Motor Function Classification System (GMFCS) by Palisano et al. [2] (Figure 1).

During the last 20 years, a lot of attention has been paid to hip issues in patients with CP. Children severely affected (in particular GMFCS level IV and V) frequently develop hip dislocations. This impairs function, especially sitting and daily care, it causes pain, and as a severe consequence, it limits quality of life. Therefore, several countries have established hip surveillance programs. A traffic light system classifies the hip (TLS-Hip) according to the radiographic findings as ”ok” (green), “at risk, monitor” (yellow) and “problem, special therapeutic intervention recommended” (red). In several countries, the implementation of the surveillance program leads to early therapeutical interventions and therewith a dramatic reduction of hip dislocations from 8% to 0.5% [3,4,5,6].

**Figure 2:**
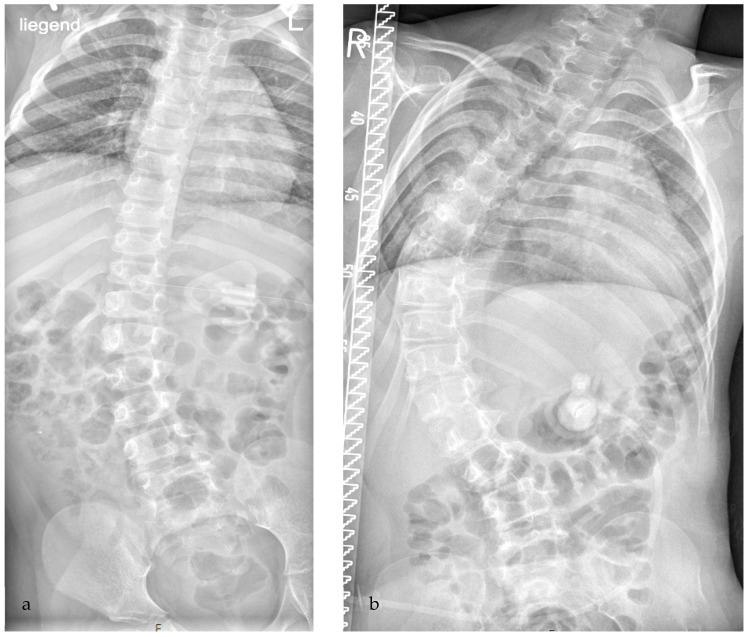
Development of severe scoliosis in a girl with CP, GMFCS level V. (**a**) At the age of 6 yrs, moderate scoliosis of 27° according to Cobb was detected; no referral to an orthopedic physician was initiated for treatment. (**b**) At the age of 10 yrs, she was first seen by an orthopedic surgeon with severe progressive scoliosis of 73°; at that point, conservative treatment is very debatable and a surgical intervention will be necessary to preserve quality of life.

However, there are not only hip problems which might develop during growth and should be addressed, there are also spinal problems which might occur in CP children. Unfortunately, at present there is only a limited focus by clinicians and physiotherapists, despite the high frequency of these conditions and the severity of impact on patients’ quality of life. The overall incidence of scoliosis is reported as between 20% and 41% in several studies [7,8,9,10]. Rates of scoliosis further increase with age and GMFCS level: at the age of 10 years, moderate or severe scoliosis has been observed in approximately 1% of the children at GMFCS level I. This rate increases to 2.5% at GMFCS III, 10% at GMFCS IV and 30% at GMFCS V; at the age of 20 years, the corresponding percentages are 5%, 30%, 45% and 80%, respectively [8]. The cause is not entirely clear, but thought to be a combination of spasticity, muscle weakness, impaired muscle control as well as asymmetric muscle tone [8,11]. The deformity is typically progressive, especially during the pubertal growth spurt but also after growth arrest [12]. Severe scoliosis may cause additional motor dysfunction, sitting and transfer problems, compromise pulmonary function and induce pain with reduced quality of life. Finally, associated respiratory insufficiency may be life-threatening. Therefore, early diagnosis and treatment is of fundamental importance to prevent severe scoliosis and to maintain quality of life, especially in the at risk groups GMFCS level IV and V (Figure 2) [8,13,14].

**Figure 3:**
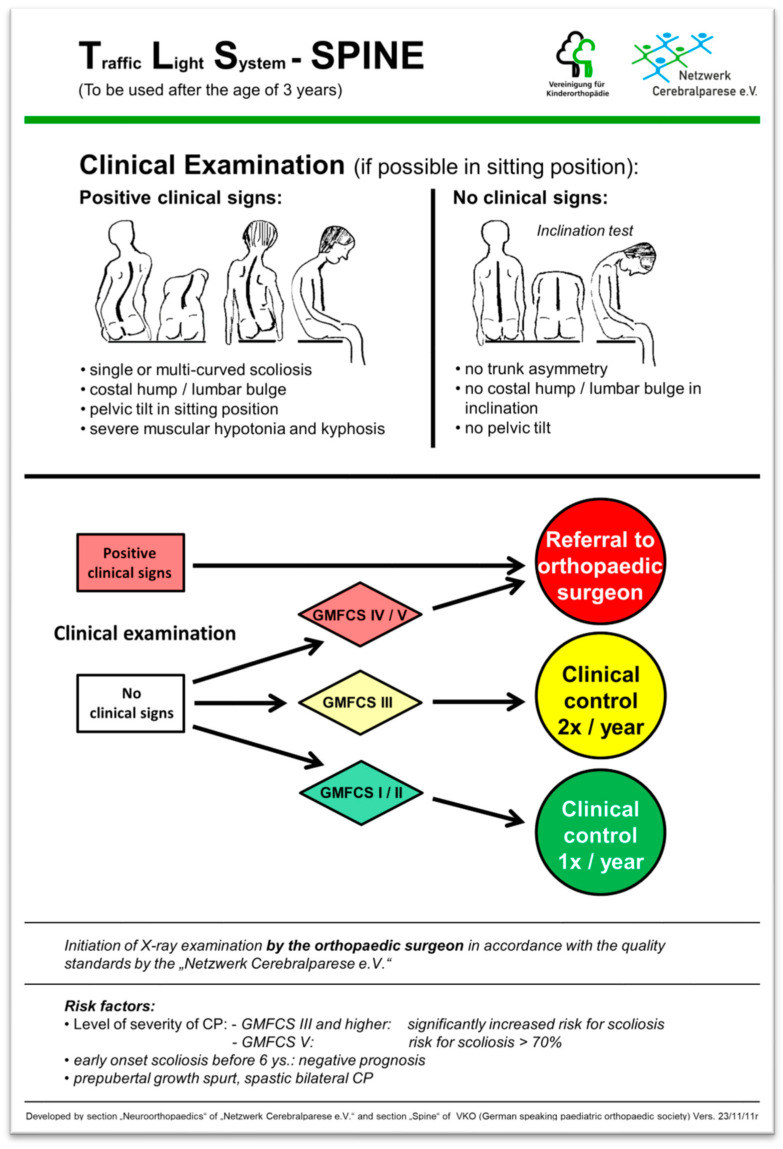
TLS-Spine (updated version considering the results of the present study). Patients are examined from the back in sitting position, an inclination test is performed; the deformity of the spine as well as the position of the pelvis are observed and graded as “normal” or “pathologic”. Based on this graduation and the assignment to a GMFCS level, patients can be classified as “green”, “yellow” or “red”. Note: all GMFCS level III patients have to be classified at least as “yellow” and all GMFCS level IV and V patients as “red”—independently of the clinical findings.

The current lack of surveillance programs in Germany leads to limited clinical awareness of spinal deformities by pediatricians and physiotherapists treating CP patients—with a negative impact on early treatment and quality of life. In response to these issues, a group of experienced pediatric orthopedic clinicians developed a traffic light system for spinal deformity classifications (TLS-Spine) in collaboration with the German speaking Society for Children’s Orthopaedics (“Vereinigung fuer Kinderorthopaedie” VKO) [15] (Figure 3). The aim was to give pediatricians and physiotherapists an instrument that is simple and quick to use in early detection of spinal problems. The tool enables clinicians to classify patients according to traffic light colors corresponding with increasing severity. The scale is based on the criteria of “functional level according to GMFCS” and “result of clinical examination”:

The classification for patients is as follows:

Green: no clinical signs of a spinal deformity, low risk factors for the development of a spinal deformity; clinical re-examination after 1 year.

Yellow: no clinical signs of a spinal deformity; however, due to increased risk of scoliosis for GMFCS level III patients, a clinical re-examination should be performed after 6 months.

Red: clinical indicators point to spinal deformity and/or very high risk for development of spinal deformity due to GMFCS level IV or V; the patient should be promptly referred to a pediatric orthopedic clinician or an orthopedic clinician specialized in neuromuscular spinal deformities.

Although personal feedback from pediatricians and physiotherapists was overall positive, this study represents the first structured evaluation of the TLS-Spine tool in clinical practice. The primary objective was to evaluate its practical value in daily routine for pediatricians and physiotherapists to manage their patients. A further aspect was whether there are differences in how pediatricians and physiotherapists perceive and apply the tool. By systematically collecting and analyzing user experiences, this study provides new insights into the tool’s usability.

## 2. Materials and Methods

### 2.1. Peer Review Group

In order to establish a professional evaluation mechanism for the TLS-Spine scale, a review group was recruited by announcements made during several national meetings, conferences and symposiums related to neuropediatric subjects. Inclusion criteria as a reviewer were 1. being a professional physiotherapist or pediatrician, including neuropediatrician, and 2. caring for CP patients as a part of their consultation and therapeutic practice. After inclusion, reviewers received general information about the project, special information about the application of the TLS-Spine in CP patients, a copy of the TLS-Spine (Figure 3), a survey sheet as a protected file for print and a questionnaire to be completed after at least 10 applications in patients had been performed.

### 2.2. Assessment Process

All reviewers were asked to apply the TLS-Spine to sample groups including a minimum of 10 CP patients aged at least 3 years and to fill out one survey sheet per patient providing individual information on patient’s age, GMFCS level and classification according to the TLS-Spine. The information about the GMFCS level of the patient and TLS-Spine classification to a group allowed a plausibility check, e.g., a GMFCS level III patient classified as “green” in the TLS-Spine indicated that the reviewer did not assign the patient correctly in that case.

Additionally, the reviewers were requested to complete a questionnaire with 9 items when they had applied the TLS-Spine in at least 10 patients. This questionnaire should give the reviewers the opportunity to give structured feedback on the usefulness and applicability of the TLS-Spine; a rating system with a scale from 1 to 6—“1” meaning complete agreement, “6” meaning complete disagreement was used (Table 2). Moreover, reviewers had an option to comment in a free-text field on the TLS-Spine. Finally, reviewers had to provide information regarding their professional experience in caring for CP patients as well as knowledge of the TLS-Hip.

During the pre-study phase, all nine authors—each working at a different medical center—conducted a preliminary evaluation by testing the use of the TLS-Spine and the questionnaire with colleagues, including physicians and physiotherapists specializing in cerebral palsy. The feedback gathered during this phase led to modifications of the questionnaire, which were subsequently incorporated into the study.

### 2.3. Data Analysis

First, each survey sheet was checked for plausibility as a quality check. The failure rate of each reviewer was calculated (%). Characteristics of the reviewers were evaluated and differences between physicians and physiotherapists statistically analyzed by the Fisher’s exact test and the Mann–Whitney U test.

The rating of the TLS-Spine by the reviewers was based on a questionnaire. Results are presented as median and minimum/maximum. Statistical significance between physicians and physiotherapists was calculated by the Mann–Whitney U test. A *p*-value < 0.05 was considered significant. The statistical data processing was carried out with SPSS Statistics 19.0.

The study was approved by the ethic committee of the Medical Faculty of the Heinrich Heine University, Duesseldorf.

## 3. Results

### 3.1. Reviewer Characteristics

For evaluation, 32 physicians and 16 physiotherapists from 29 medical centers could be recruited, who submitted 48 reviews. All reviewers completed the questionnaire based on 537 examinations using the TLS-Spine in CP patients—on average 11.2 patients per reviewer. A total of 517 of 537 survey sheets pertaining to the TLS-Spine were filled out and classified correctly (96.3%). Of the 20 cases identified as misclassified, 15 showed a discrepancy between the GMFCS level and the assigned traffic light color—8 patients with GMFCS level IV or V were incorrectly classified as “green” or “yellow” in the TLS-Spine system—and 7 patients with GMFCS level III were classified as “green”; additionally, in 5 cases, the classified child was under the age of 3.

Relevant information concerning the reviewers is described in Table 1. Between physicians and physiotherapists, there were no statistically significant differences in relation to failure rate in coding the TLS-Spine, duration of professional practice, experience with CP patients and experience with TLS-Hip.

All of the 32 physicians had completed their specialist training in pediatrics and/or neuropediatrics. In total, 87.5% reported long-term experience with cerebral palsy and 9.4% reported moderate length experience. One colleague did not answer this question. Out of all the physicians, the rate of incorrect application of the TLS-Spine was 3.5%. Overall, 67.7% had experience with the TLS-Hip in the past. Of the 16 physiotherapists involved in the review, 87% had completed additional training on neuro-physical disorders. A total of 81.3% mentioned long-time experience with cerebral palsy and 12.5% reported moderate experience. One respondent did not answer the question. The rate of incorrect coding of the TLS-Spine was 4.1%. Overall, 42,9% used the TLS-Hip in the past. Reviewers with previous experience with the TLS-Hip tended to show a lower rate of incorrect coding than those with no experience, which was not statistically significant.

### 3.2. Evaluation of the TLS-Spine Application

**Figure 4:**
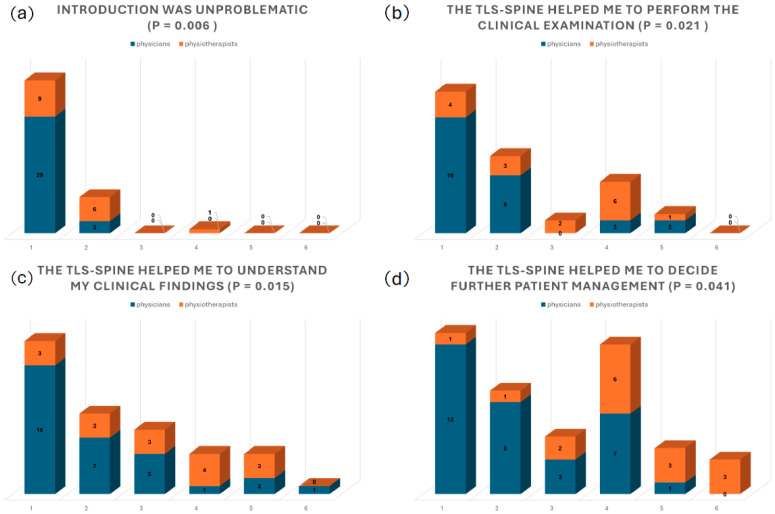
Distributions of the significant answers to (**a**) “Introduction of TLS-spine in daily clinical work was unproblematic”, (**b**) “The TLS-spine helped me to perform the clinical examination”, (**c**) “The TLS-spine helped me to understand my clinical findings” and (**d**) “The TLS-spine helped me to decide further management of the patient”. The x-axis shows the rating of the respondents on a scale from 1 to 6—“1” meaning complete agreement and “6” meaning complete disagreement; the y-axis shows the frequency.

The analysis of the questionnaire (Table 2) which was answered by the reviewers demonstrated that the introduction and application of the TLS-Spine in daily clinical routine was well accepted by respondents. Overall, the introduction of the TLS-Spine in daily clinical work was without any problems, working with it was straightforward and not time consuming, it helped to perform the clinical examination, to understand the findings and to decide on the further management of the patients; for several respondents, the TLS-Spine initiated new reflections on patient management (Figure 4).

The comparison between physicians and physiotherapists showed statistically significantly that in particular physicians indicated positive impact of the TLS-Spine in performing clinical examinations, understanding the clinical findings and in decision making on further patient management (Table 2, Figure 4).

In general, physicians as well as physiotherapists reported a positive impression of the TLS-Spine (Table 2).

The open comments gave a wide spectrum of feedback—from “we use it for any CP patient in our institution”, “the TLS-Spine increases the awareness for spinal deformities” up to “need for special training”, “time period for orthopaedic examination in case of ‘red’ missing”, “TLS-Spine not sufficiently detailed, GMFCS IV and V are always accompanied by deformities—therefore, what for?”

## 4. Discussion

To raise awareness of spinal deformities in CP children, a tool based on a traffic light system was developed for pediatricians and physiotherapists involved in their care. The aim of this study was to evaluate the applicability of the tool in everyday clinical practice. Based on at least 10 uses, the reviewers—pediatricians and physiotherapists—reported that applying the TLS-Spine tool posed no difficulties; they found it straightforward to use, not time consuming and helpful in conducting clinical examinations, interpreting findings and making decisions about further patient management. This study represents the first systematic evaluation of user experiences with the TLS-Spine.

Spinal deformities—especially scoliosis—are an underestimated concern in the care of patients with cerebral palsy, especially for patients functioning at the GMFCS level IV and V. Otherwise, it is not explainable that a relevant number of patients see an orthopedic specialist the first time when the spinal deformity is already severe. Severe scoliosis will lead amongst others to additional motor dysfunction, cardio-pulmonal insufficiency, pain and overall, to a significant reduction of quality of life. Surgical interventions to correct the deformity are challenging and do have a high risk for complications [13,16,17]. Therefore, it is vital to increase awareness and early detection of spinal deformities in cerebral palsy patients. This enables an early referral to an orthopedic specialist where the most appropriate treatment decision can be made, like surgery at the appropriate time, before severe high-risk surgery is needed. In Sweden, the clinical examination of the spine is part of a CP surveillance program (https://cpup.se/) and therefore allows early identification of curve progression and initiation of conservative or surgical interventions.

Several risk factors have been identified for developing scoliosis in CP patients [10,12,13,18]. Based on the data of the Swedish CP registry, Pettersson et al. developed a risk score to predict the individual risk for a 5-year-old child with CP to develop a severe scoliosis before the age of 16 years [19]. The analysis revealed four predictors for scoliosis: female sex, GMFCS levels IV and V, epilepsy and limited knee extension. The predictive ability of the score was high, with an area under the receiver operating characteristics curve of 0.87 (95% CI 0.84–0.91), indicating a high accuracy in differentiating between high- and low-risk individuals.

Unfortunately, in Germany and many countries worldwide, there is no comparable surveillance program. Motivated by the positive effect of the TLS-Hip and the negative impact of scoliosis on the patient’s physical condition and quality of life, a group of experienced pediatric orthopedic surgeons with specialist experience in neuroorthopedics developed a new tool—called TLS-Spine. It is designed especially for non-orthopedic physicians and physiotherapists, who are involved in the care of CP patients, to increase their awareness for spinal deformities. Requirements for wide acceptance of such a tool include ease of access and applicability as well as provision of specific information to further address issues identified.

The objective of this study was to evaluate the TLS-Spine in relation to the requirements outlined. The overall assessment indicated that the target group (pediatricians and physiotherapists) rated the TLS-Spine as easy to implement and apply within existing daily workloads without additional strains on time. Especially, physicians rated the TLS-Spine positive in respect to performing the clinical examination, understanding the clinical findings and drawing conclusions for further management of the patient. This might be since physiotherapists are well trained in performing a clinical examination, so they do not need additional support; they are not responsible for making decisions on treatment concepts, so they probably do not benefit that much from this tool. But on the other hand, they also rated it as a useful tool in daily clinical routine—like the physicians.

Consistency checks revealed that 3.7% of the patients were classified incorrectly. This failure rate is supposed to be quite low and acceptable.

As a consequence of this study and the feedback of the reviewers, the team has already modified the TLS-Spine and improved its design with a clearer pathway in order to avoid misunderstandings in terms of therapeutic consequences. The updated version of the TLS-Spine is shown in Figure 3.

In Germany, the TLS-Spine was graded as very helpful by the neuropediatricians for their daily routine in CP patients. As a consequence, the German Society of Social Pediatrics and Adolescent Medicine (DGSPJ), German Society of Neuropediatrics (GNP) and German-speaking Society for Children’s Orthopedics (VKO) accepted the use of the TLS-Spine as a quality criterion for institutions caring for CP patients. Therefore, we can recommend this tool as a guideline for pediatricians and physiotherapists of other countries where no specific surveillance program exists.

Several limitations to the evaluation process have been identified. First, the selection process of the reviewers was not standardized and the ratio of physicians to physiotherapists was uneven, yielding a surplus of physicians in the review group. Second, the inter- and intrarater variability was not assessed although this aspect has no relation to the main research question. Third, there was no special training module in place for the reviewers apart from the guidance provided by the TLS-Spine itself and the information package. The team deemed specific training to be unnecessary as the application of the instrument ought to be self-explanatory. However, the lack of a training module was an additional reason to modify the design of the TLS-Spine and still it is considered to implement a training program. Forth, a test/retest-study to assess reliability was not conducted. Fifth, the efficacy of the application of the TLS-Spine for patient care was not assessed. Sixth, the TLS-Spine was not formally adapted into English.

Future studies should focus on validating the reliability of the TLS-Spine and assessing its effectiveness in CP children. Specifically, research should investigate its potential to support early intervention for spinal deformities, prevent the progression to severe deformities and evaluate its impact on patients’ quality of life.

## 5. Conclusions

The TLS-Spine is an easy to apply tool for increasing awareness of spinal deformities in pediatricians and physiotherapists caring for CP patients. The tool may increase rates of early referrals to orthopedic specialists who do have extended qualified clinical and radiographic diagnostic options and may implement early treatments. For patients, this has the potential for improving long-term clinical outcomes as well as increasing quality of life.

## Figures and Tables

**Table 1:** Characteristics of the reviewers.

Criteria	Physicians	Physiotherapists	Total	*p*
Reviewers	32	16	48	
TLS-Spine survey sheets	367	170	537	
Incorrect coded TLS-Spine (n)	3.5% (13)	4.1% (7)	3.7% (20)	n.s.
Professional practice since graduation—median years (range)	17.5 (2–40)	17.5 (6–32)	17.5 (2–40)	n.s.
Self-reported experience with CP big (n) moderate (n) small (n)	87.5% (28) 9.4% (3) 0%	81.3% (13) 12.5% (2) 0%	85.4% (41) 10.4% (41) 0%	n.s.
Previous work with TLS-Hip (n)	67.7% (21/31)	42.9% (6/14)	60.0% (27/45)	n.s.

**Table 2:** Results of the questionnaire.

Criteria	Physicians n = 32		Physiotherapists n = 16		Total n = 48		
	Median	Min–Max	Median	Min–Max	Median	Min–Max	*p*
Introduction of TLS-Spine in daily clinical work was unproblematic	1	1–2	1	1–4	1	1–4	0.006
Working with the TLS-Spine was straight forward	1	1–3	1	1–3	1	1–3	n.s.
The TLS-Spine helped me to perform the clinical examination	1	1–6	3	1–5	2	1–6	0.021
The TLS-Spine helped me to understand my clinical findings	1.5	1–6	3	1–5	2	1–6	0.015
The TLS-Spine helped me to decide further management of the patient	2	1–4	3	1–5	2	1–5	0.041
The TLS-Spine is useful in my daily clinical routine	1	1–2	1	1–4	1	1–4	n.s.
The usage of the TLS-Spine is not time-consuming in daily routine	1	1–4	1	1–4	1	1–4	n.s.
The TLS-Spine initiated new reflections on patient management	4	1–6	4	1–4	4	1–6	n.s.
General impression	2	1–4	2	1–5	2	1–5	n.s.

Answers by the respondents on a scale from 1 to 6—“1” meaning complete agreement and “6”meaning complete disagreement.

## Data Availability

Data available on request.

## Abbreviations

The following abbreviations are used in this manuscript:

CP	Cerebral palsy
GMFCS	Gross motor function classification system
TLS	Traffic light system
